# Conservative Management of a Dentigerous Cyst Associated with an Impacted Mandibular Second Premolar in Mixed

**DOI:** 10.5681/joddd.2009.025

**Published:** 2009-09-16

**Authors:** Pradeep Kumar Mohapatra, Namita Joshi

**Affiliations:** ^1^Orthodontist, Dental Department, Hamad Medical Corporation, Doha, Qatar; ^2^Specialist, Dental Department, Community Medical Center, Al-Khoor, Qatar

**Keywords:** Dentigerous cyst, impacted bicuspid, mixed dentition, odontogenic cyst, oral surgery

## Abstract

Dentigerous cysts, commonly encountered in the practice of dentistry, are benign odontogenic cysts associated with crowns of unerupted and/or impacted permanent teeth. They frequently occur during the second and third decades of life. Treat-ment modalities range from enucleation to marsupialization, which may be influenced by the age of the patient, severity of impaction, and root form of associated tooth/teeth. The purpose of this report is to describe the successful outcome of con-servative surgical management of a large dentigerous cyst associated with an impacted mandibular second premolar in a young patient.

## Introduction

Dentigerous cyst or follicular cyst is an odontogenic cyst associated with the crown of an impacted, embedded, unerupted or developing tooth. The cyst which encloses the crown of an unerupted tooth is attached to the cervical region of the tooth. It is the second most common type of odontogenic cysts accounting for 14% to 24% of all jaw cysts.^1 , 2^ Although these cysts occur more frequently during second and third decades of life, they can also be found in children and adolescents in the mixed dentition stage.^1 - 3^ Males are slightly more likely to develop dentigerous cysts than females.^1 , 4^ Most frequently, they are found around the crowns of mandibular third molars, followed by maxillary canines and maxillary third molars. Maxillary and mandibular premolars have also been associated with dentigerous cysts. ^1 - 7^ Dentigerous cysts have also been reported in association with impacted deciduous teeth.^8 , 9^

Clinically, patients with dentigerous cysts are generally asymptomatic. They are often described as an incidental radiographic finding on routine radiographs or when films are obtained to determine why a tooth has failed to erupt or when an acute inflammation or infective exacerbation occurs.^10^ The usual radiographic feature is characterized by a symmetric, well-defined, usually unilocular radiolucent lesion surrounding the crown of an unerupted tooth. Generally there is a distinct, dense periphery of reactive bone (condensing osteitis) with a radiolucent center. These cysts can also manifest as multilocular entities and occasionally may be associated with resorption of the roots of adjacent erupted teeth.^11 - 13^

Dentigerous cysts are generally treated by surgical means. The most common surgical modalities used are total enucleation,^2^ marsupialization,^4, 5^ and decompression of the cyst via fenestration.^6^

This case study describes a conservative surgical approach combined with routine orthodontic treatment of a dentigerous cyst associated with a mandibular second premolar in an adolescent.

## Case report

An 11-year-old female was referred by her dentist in Community Medical Center to the Orthodontic Section, Department of Dentistry, Hamad Medical Corporation, Doha, Qatar, with the chief complaint of a swelling on the left side of her lower jaw since three months earlier. The swelling had been growing slowly over the period and was associated with no pain or discharge. The overall general physical health of the patient was good with nonspecific general medical history, without any contraindication to dental treatment.

**Figure 1:**
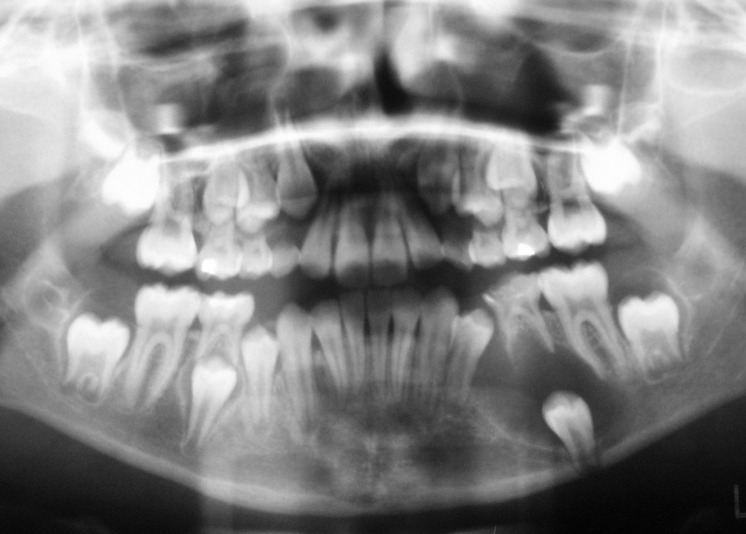
Pretreatment panoramic radiograph showing a large, well-circumscribed, unilocular radiolucent lesion in the body of the mandible on the left side with displaced second premolar at its lower border. The root has developed approximately half of its original length.

The extraoral examination revealed a symmetrical orthognathic facial profile with no signs of neurological deficit in the lower half of the face. There was no sign of any regional lymphadenopathy. Intraoral examination revealed a mixed stage of dentition, bilateral Class I molar relationships and Class I incisors. A hard, non-tender, non-fluctuant swelling of 2.5 × 2 cm was evident in the lower left vestibule, extending from the distal surface of the left permanent canine to the distal surface of ipsilateral first permanent molar. The swelling was associated with expansion of buccal and lingual cortical plates and covered by healthy-appearing and freely-moving mucosa. The teeth adjacent to the swelling were quite firm and not associated with any decay. A panoramic radiographic examination (OPG) revealed the presence of all the permanent teeth without any decayed or supernumerary teeth. There was a well-circumscribed unilocular radiolucent lesion in the body of the mandible on the left side, which was associated with the crown of a vertically impacted second premolar. The root of the impacted second bicuspid was developed approximately up to half of its usual length and the apex was quite wide open. The cystic structure appeared to have originated from the second bicuspid with inferior and distal displacement of the same tooth. The corresponding deciduous tooth (second molar) was still present with normal crown and roots (Figure 1).

A clinical diagnosis of a dentigerous cyst involving the crown of the impacted left mandibular second bicuspid was made with the differential diagnosis of an inflammatory cyst, a keratocyst and a unilocular ameloblastoma.

### Treatment

*Aims and objectives*

Considering the age of the patient, her occlusal status, size of the cyst, position, and developmental stage of the root of the involved tooth a conservative treatment modality was decided upon. The main objectives of the treatment were clinical and radiographic elimination of the pathologic entity and to bring the involved permanent tooth into its proper position.

*Treatment plan*

- Extraction of the left mandibular second deciduous molar and decompression of the cyst through the extraction socket.
- Histopathologic examination of the cystic lining.
- Trans-lingual arch to hold the permanent first molars bilaterally in their current position and to maintain the space for unerupted left bicuspids.
- Follow-up of the progress of eruption of the impacted second bicuspid with periodic radiographs.
- Fitting of a fixed orthodontic appliance for final alignment of teeth in due course if needed.

*Treatment progress*

**Figure 2:**
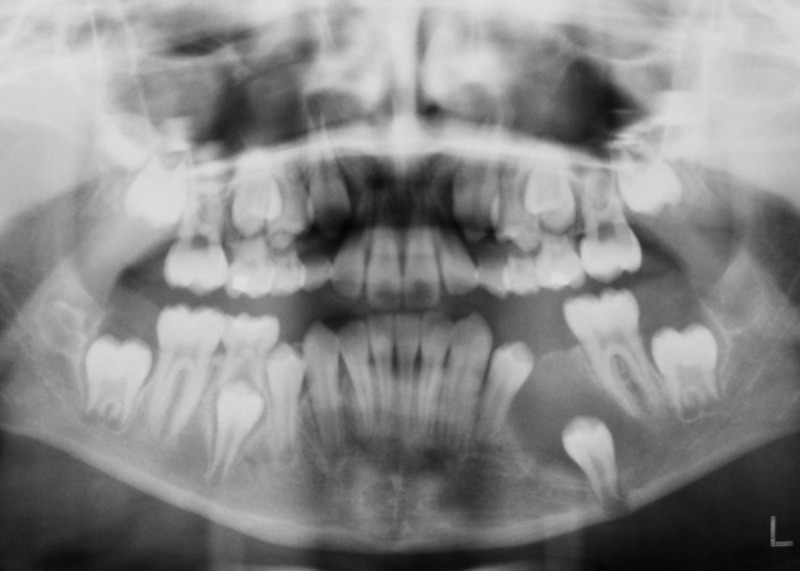
Panoramic radiograph, two weeks after surgery.

The left second deciduous molar was extracted under local anesthesia (2% Lidocaine with 1:100,000 Epinephrine) and the socket was used to establish a communication between the cyst cavity and the oral cavity. An incisional biopsy was obtained from the cyst wall for histopathologic examination, which confirmed the initial diagnosis of a dentigerous cyst without evidence of any dysplastic changes. A BIPP (bismuth iodoform paraffin paste) gauze pack was inserted into the cyst cavity and secured with a suture. One week after surgery, the pack was removed and repacking was done with another BIPP gauze pack, which was kept in place for another week. Two weeks after surgery, the patient was sent back to an orthodontist for the fabrication of trans-lingual arch and for further follow-up (Figure 2).

**Figure 3:**
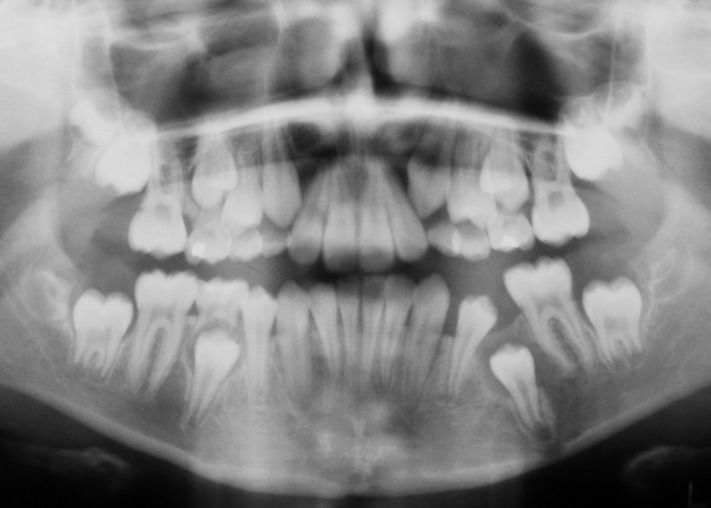
Panoramic radiograph, eight months after surgery, showing eruption of second premolar up to the level of contralateral premolar and bony fill into the cyst cavity. Note the drift of adjacent teeth into the extraction space and insufficient space for left second premolar.

**Figure 4:**
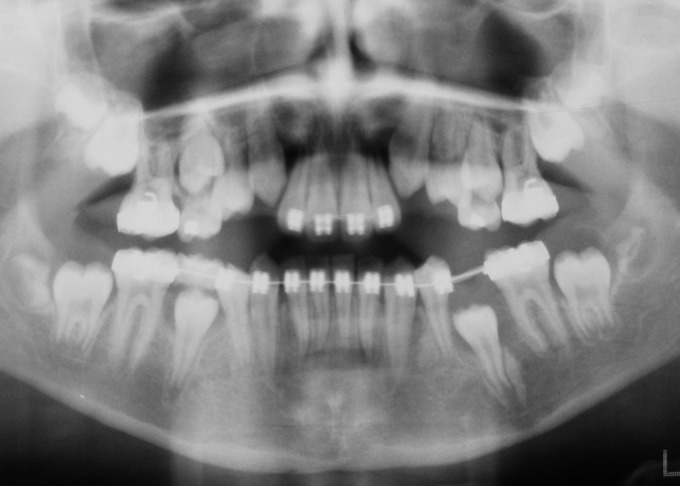
Panoramic radiograph, sixteen months after surgery, showing eruption of second premolar with normal root morphology and further bony fill of the cyst cavity as evidenced by increased radiopacity. Note the open space for second premolar.

**Figure 5:**
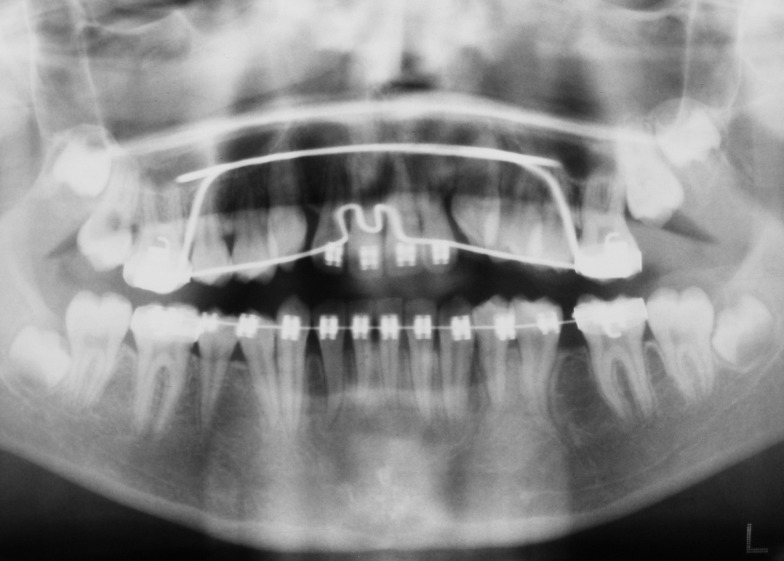
Panoramic radiograph, 30 months after surgery, showing normal alignment of lower left second premolar and normal trabecular bone at the cyst site.

**Figure 6:**
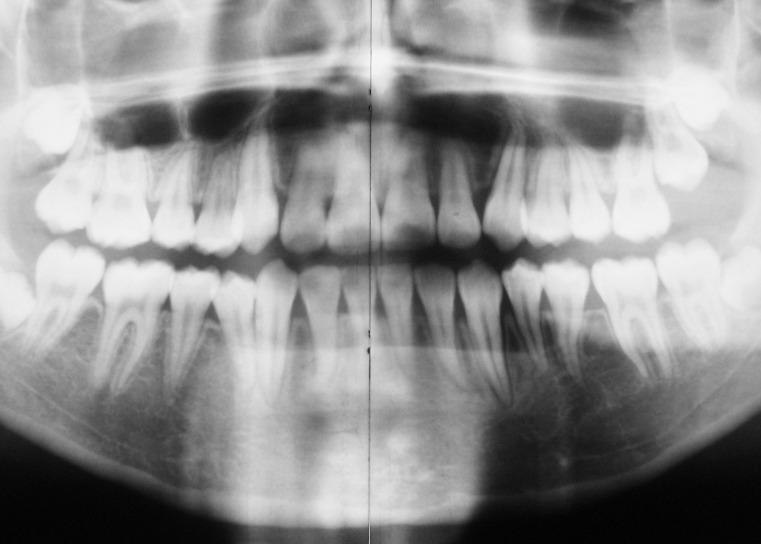
Post-treatment panoramic radiograph, 37 months after initial surgery, shows complete radiologic healing of the cystic lesion with normal trabecular bone and normal alignment of the left mandibular second premolar.

However, the patient could not return for further follow-up and treatment as she was out of the country. She reported eight months after the initial surgery and at this stage the clinical examination revealed inadequate space for the unerupted second premolar. The panoramic view (OPG) showed a favorable change in the position of the impacted premolar with increased

radiopacity of cystic lesion, suggesting osteogenesis (Figure 3). A fixed orthodontic appliance was fitted in the lower arch without further delay with the aim of reopening the lost space for the impacted premolar and preventing mesial migration of the permanent first molar. The space was opened with the help of an open coil spring and the permanent first molar was held in position by a mesial stop in the archwire. The impacted premolar was monitored for its eruption by periodic panoramic radiographs (Figure 4). After twenty-four months, the impacted premolar erupted spontaneously. The tooth was bonded and engaged into the archwire when it erupted up to bondable crown height. After a few weeks it was aligned to its normal occlusal position (in relation to its adjacent, contralateral and opposing teeth) (Figure 5). It took a total of 37 months to finish the treatment. The final panoramic view which was taken just after debonding shows complete radiologic healing of the cystic lesion with normal trabecular bone and normal alignment of the impacted left mandibular second premolar (Figure 6).

## Discussion

The aim of treatment for dentigerous cysts is complete elimination of pathology and maintenance of dentition with minimal surgical intervention. Recently-defined criteria for selecting the treatment modality refer to the cyst size and location of the cyst, patient age, dentition involved, stage of root development, position of the involved tooth in the jaw and its relation to the adjacent teeth, and involvement of adjacent vital structures.^14^

Surgical treatment of dentigerous cysts usually includes enucleation of the lesion along with the removal of associated teeth.^2 , 6 , 8 , 14^ This approach is favored in cases involving impaction of a single tooth, such as a wisdom tooth in an adult, which has no function; however, it is not often in the patient’s best interests. In particular, extractions of associated teeth in children may have functional, esthetic, and psychological consequences. Thus a conservative surgical approach was chosen for this patient, which consisted of the removal of one deciduous tooth and an incisional biopsy for histological examination, considered essential to confirm the diagnosis. Many authors have emphasized the importance of maintaining the opening between the cyst and the oral cavity artificially not only to heal the cystic lesion but also to prevent the formation of a fibrous scar which can impair eruption of the involved teeth.^4 , 5 , 7 , 10^ However, in this case no such attempt was made to maintain the patency of the cyst opening into the oral cavity except for keeping the surgical pack in place for only two weeks. This finding supports the hypothesis that simple decompression of the cyst and further root development would allow for the spontaneous relocation of the associated tooth.

According to Miyawaki et al,^7^ the optimal timing for the initiation of surgical treatment of a cyst-associated tooth is when the tooth has the ability to erupt. Radiographically the roots of these teeth should show at least 2/3 of root formation with an open apex. Although optimal timing correlates with the 2/3 of root development, in this case the radiograph showed approximately 1/2 of root development. The decision was made to administer treatment at this stage considering the initial position of the involved tooth in relation to the lower border of the mandible and to allow for further root development by an early release of intra-cystic pressure.

It has also been well-documented by many authors in different case studies that complex orthodontic treatment can be avoided by maintaining the space in the arch for underlying involved tooth.^5 , 6^ However in this case an early initiation of orthodontic treatment was essential as there was inadequate space for the impacted second premolar due to drifting of adjacent teeth into the extraction site of the second deciduous molar.

It took a total of 37 months to finish the treatment, which emphasizes the importance of close supervision by the operators. The result also highlights the potential for healing of the cystic lesion after its decompression, particularly in young children. As revealed by the panoramic view, there was complete radiologic healing of the cystic lesion, including the filling of cystic cavity with normal trabecular bone at the end of the treatment (Figure 6).

## Conclusion

This case report presents a number of points which are noteworthy:

1. A simple and conservative surgical approach should be preferred for a large dentigerous cyst in an adolescent in the mixed dentition period. This not only preserves the function and esthetic values but also prevents the child from psycho-social trauma due to tooth loss.
2. The capacity to regenerate bone is greater among children than among adults and teeth with open apices have great eruptive potential. Thus, large dentigerous cysts in children can be treated differently and conservative treatment with tooth preservation should always be considered.
3. This case report also provides a commentary on the optimal timing to initiate surgical treatment of a cyst-associated tooth. It supports the concept of initiation of treatment when radiographic analysis of the roots and apex of these teeth shows at least 1/2 - 2/3 of root formation with an open apex.
4. A panoramic (OPG) film is a common and reliable tool for the diagnosis and assessment of progress in healing of a mandibular dentigerous cyst along with the change in the position of the associated tooth.

However, this conservative approach does require close cooperation on the part of both the patient and the dental practitioners in order to monitor the healing of the lesion and change in the position of the involved tooth. The result can be the elimination of pathology and maintenance of the dentition.
